# Operationalizing a Hub‐and‐Spoke Telemedicine Model for Mpox Surveillance in a High‐Alert, Zero‐Prevalence Setting: An Observational Study, Real‐World Experience From Iran

**DOI:** 10.1155/ipid/9955516

**Published:** 2026-01-31

**Authors:** Owrang Eilami, Nader Eilami, Alireza Heiran, Mehdi Nejat, Mehrab Sayadi

**Affiliations:** ^1^ HIV/AIDS Research Center, Institute of Health, Shiraz University of Medical Sciences, Shiraz, Iran, sums.ac.ir; ^2^ Applied Economics, Wuppertal University, Wuppertal, Germany; ^3^ Health System Research Department, Vice Chancellor of Health Affairs, Shiraz University of Medical Sciences, Shiraz, Iran, sums.ac.ir; ^4^ Center for Communicable Diseases Control, Vice Chancellor of Health Affairs, Shiraz University of Medical Science, Shiraz, Iran, sums.ac.ir; ^5^ Vice Chancellor for Health Affairs, Shiraz University of Medical Sciences, Shiraz, Iran, sums.ac.ir; ^6^ Cardiovascular Research Center, Shiraz University of Medical Sciences, Shiraz, Iran, sums.ac.ir

**Keywords:** mHealth, monkeypox, monkeypox virus, Mpox, telecommunications, telehealth, telemedicine, virtual medicine

## Abstract

**Background:**

The global monkeypox (Mpox) outbreak prompted heightened surveillance in regions with significant travel links. Fars Province, Iran, implemented a structured telemedicine response to manage patients presenting with Mpox‐like symptoms.

**Objectives:**

This study aimed to describe and evaluate the impact of a tiered, hub‐and‐spoke telemedicine model on the triage, differential diagnosis, and cost efficiency of managing suspected Mpox cases in a setting with no confirmed Mpox.

**Materials and Methods:**

In this observational study conducted from August 27 to September 22, 2024, 150 patients presenting with fever and vesiculopustular rash across Fars Province were managed via a mandated protocol. Cases unresolved by local physicians were escalated via asynchronous (store‐and‐forward) WhatsApp consultations to a central specialist hub. Diagnostic testing, including Orthopoxvirus (Mpox) PCR, varicella zoster virus PCR, and herpes simplex virus testing, was performed based on telemedicine triage. A cost–consequence analysis compared the implemented pathway to a hypothetical standard referral scenario.

**Results:**

Among 150 teleconsultations, 28 patients (18.7%) were triaged as high suspicion for Mpox; three had relevant international travel history. No Mpox cases were confirmed. Final diagnoses were varicella (56.7%), herpes zoster (27.3%), herpes simplex (8.0%), and other conditions (8.0%). The telemedicine model prevented 122 (81.3%) unnecessary in‐person specialist referrals. The median consultation response time was 95 min. The cost analysis showed a 76% reduction in direct costs, saving an estimated 1,087,500,000 Iranian Rials compared to standard care.

**Conclusion:**

A tiered telemedicine model proved effective for outbreak preparedness, enabling rapid expert triage, accurate differential diagnosis, and significant resource savings in a high‐alert, zero‐prevalence setting. This approach might yield a scalable blueprint for managing future alerts of emerging infectious diseases with cutaneous manifestations.

## 1. Introduction

Monkeypox (Mpox), caused by the Mpox virus, is a zoonotic disease characterized by a prodrome of fever, malaise, and lymphadenopathy, typically followed by a distinctive rash, 1–4 days after fever onset. The cutaneous lesions usually progress through sequential stages—macules, papules, vesicles, and pustules—before crusting over. The rash distribution can be widespread or localized, frequently involving the face, extremities, genitalia, and mouth/throat. Diagnosis relies on clinical suspicion confirmed by nucleic acid amplification testing (e.g., PCR) for Orthopoxvirus. The mainstay of management is supportive (e.g., pain management, hydration, skin care, and prevention of or treatment of secondary infections), although antiviral agents such as tecovirimat may be used in severe cases [1–6].

Following decades of endemic, zoonotic transmission in Central and West Africa, characterized by a noticeable increase in reported cases and intermittent outbreaks after the cessation of smallpox vaccination [7, 8], a global outbreak of a less severe Clade IIb virus occurred in 2022, which led to a dramatic shift in epidemiology. This outbreak was characterized by rapid human‐to‐human transmission, usually linked to sexual contact networks, and extensively occurred in nonendemic regions [9, 10]. In 2024, a more virulent and transmissible Clade Ib variant emerged and spread in Africa, followed by international travel‐related cases, which subsequently led to the declaration of a public health emergency of international concern and further heightened global surveillance needs [11]. Given Iran’s status as a major travel destination, it faced a tangible threat of virus importation [12]. Although cases have been reported in neighboring countries, to date, however, only sporadic imported Mpox cases have been reported from Iran, as evidenced by a confirmed travel‐related case during the 2022 outbreak [13]. Hence, these cases and the growing international risk of importation have raised the need for preparedness and efficient triage systems to prevent local transmission. Although an effective vaccine exists [14], its limited availability in many regions, including Iran, makes nonpharmaceutical interventions essential for outbreak control.

Telehealth is broadly defined as the remote delivery of clinical services using information and communication technologies to bridge geographic separation, which has become a vital tool in infectious disease management [15, 16]. It can potentially reduce system costs through shorter interactions, reduced travel, and more efficient resource allocation [17]. Telemedicine is a key component of telehealth, and its delivery via smartphone‐enabled, store‐and‐forward (SAF; asynchronous) platforms is of interest and suited for conditions mainly characterized by cutaneous findings, such as Mpox. This modality allows for rapid sharing of clinical data in images, videos, and audios for specialist consultation, preventing unnecessary physical contact [18–21]. During outbreaks, such systems can facilitate timely triage, reduce unnecessary exposures in healthcare settings, optimize resource allocation, and maintain care continuity [22, 23]. Moreover, tele‐education—the remote training of healthcare workers using digital platforms—may further support and strengthen outbreak response through rapid dissemination of clinical guidelines and collaborative learning, which would be critical when managing emerging pathogens with which frontline providers might have limited experience [24–27].

During the 2024 global Mpox alert, the Fars Province health system in Southern Iran faced the considerable risk of imported cases due to the mass gatherings for the Ashura commemorations. That is, there was a need to efficiently triage a potential surge of patients with Mpox‐like symptoms, in addition to minimizing resource strain and preventing hospital overload. Therefore, this study aimed to report the implementation and evaluate a tiered, hub‐and‐spoke telemedicine model for the triage and management of suspected Mpox cases. We assessed its operational feasibility, diagnostic performance, and cost efficiency in a real‐world, high‐alert setting with zero confirmed Mpox prevalence.

## 2. Materials and Methods

### 2.1. Study Design and Setting

This observational study reports the real‐world implementation and outcomes of a province‐wide telemedicine triage protocol activated by the Vice Chancellor for Health Affairs of Shiraz University of Medical Sciences (SUMS), Shiraz, Iran, during the 2024 global Mpox alert. The protocol was designed to manage a surge of suspected cases following mass gatherings for the Ashura commemorations in late August 2024, which raised concerns about importation from neighboring countries with confirmed cases. The response was coordinated in collaboration with the Vice Chancellor of Health Affairs Department and Namazi Hospital, the tertiary infectious disease referral center in Fars Province. The study period was from August 27, 2024, to September 22, 2024, aligning with the official provincial alert.

### 2.2. Governance and Network Structure

**Figure 1:**
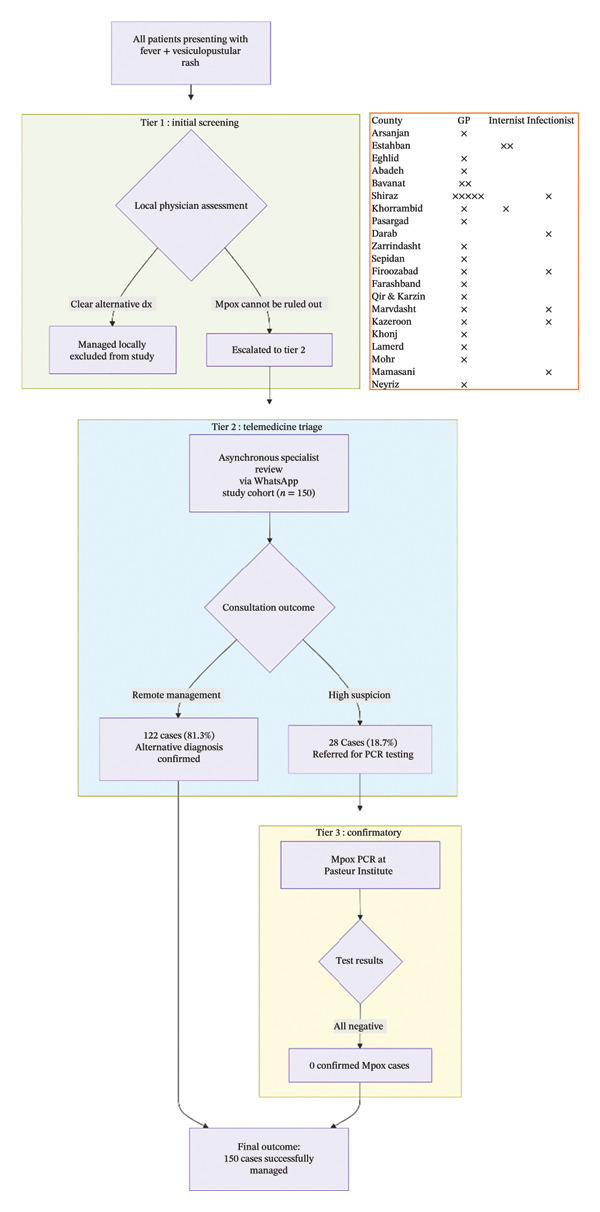
Flowchart of the three‐tiered telemedicine protocol for the triage and management of suspected Mpox cases in Fars Province, Iran (2024). The pathway illustrates the stepwise filtration of patients presenting with fever and rash. Tier 1 (initial screening): Local primary care physicians performed the first assessment. Distribution of the 32 referring physicians in the telemedicine network by specialty is illustrated in the orange‐bordered box. The network was composed primarily of GPs based in comprehensive health centers across 21 counties, supported by internists and local infectious disease specialists. Cases with a clear alternative diagnosis were managed locally and were not part of the study cohort. Tier 2 (telemedicine triage hub): Cases where Mpox could not be ruled out (*n* = 150) were escalated for asynchronous specialist consultation via a WhatsApp‐based system. Remote review led to direct management for most patients (*n* = 122) or referral for confirmatory testing (*n* = 28). Tier 3 (confirmatory testing): High‐suspicion cases underwent PCR testing at the national reference laboratory, with all results returning negative. The protocol successfully managed all 150 escalated cases with zero confirmed Mpox infections.

Operational leadership was assigned to two appointed focal points (i.e., an attending adult infectious disease specialist and a pediatric infectious disease specialist at SUMS). They coordinated with a pre‐established network of 32 referring physicians across 21 counties within the SUMS jurisdiction (excluding the independent universities of Fasa, Jahrom, and Lar). This network consisted of general practitioners (GPs) in comprehensive health centers, internists, and local infectious disease specialists (see Figure 1 for distribution).

### 2.3. The Telemedicine Triage Protocol: A Tiered, Hub‐and‐Spoke Model

A tiered, hub‐and‐spoke consultation model was mandated as the standard public health response (Figure 1).

Any patient presenting at a primary or secondary care facility with the syndromic case definition of acute fever (> 38°C) and vesiculopustular or ulcerative skin rash was initially assessed by the local referring physician (Level 1; county‐level consultation).

At the provincial telemedicine hub (Level 2), cases that could not be confidently ruled out for Mpox by the local physician were formally escalated. The referring physician compiled a deidentified consultation package, which included the patient’s clinical history and epidemiological data—such as travel links—along with photographs or videos of the skin lesions and relevant extracts from the patient’s health record in text or photo form. This package was transmitted securely via WhatsApp to the designated focal point specialists, thereby constituting an asynchronous SAF teleconsultation. Upon review, the specialists yielded diagnostic and management feedback through voice messages or text. Their recommendations could include recommending an alternative diagnosis (e.g., disseminated herpes zoster) with supportive care instructions, requesting additional local tests such as a Tzanck smear or VZV/HSV IgM serology, or issuing a directive to collect samples for Mpox PCR testing. Verbal consent was obtained from patients for the capture and secure remote consultation of their deidentified clinical photographs.

For patients triaged as high suspicion, the focal points coordinated immediate sample collection. Samples were obtained from skin lesions with active discharge using sterile swabs at the designated SUMS Coronavirus Laboratory (SUMS Coronalab). Following a strict national protocol, these samples were then shipped with a cold chain to the Pasteur Institute of Iran in Tehran for confirmatory Orthopox virus (Mpox‐specific) PCR testing. Complex cases requiring hospitalization were referred directly to the isolation wards at Namazi Hospital.

### 2.4. Participants, Data Collection, and Final Diagnosis

All consecutive patients escalated to the provincial telemedicine hub during the study period were included in this analysis. The escalation itself served as the inclusion criterion, representing the cohort of cases that primary care could not definitively rule out. For each case, data on demographics, clinical presentation, teleconsultation notes, test results, and final diagnosis were collected. The final diagnosis was established by the focal points based on the teleconsultation and any subsequent test results. Epidemiological links for suspected cases were actively investigated via patient interviews.

### 2.5. Cost–Consequence Analysis

A cost–consequence analysis was performed from the health system perspective. The implemented tele‐triage pathway was compared to a hypothetical standard referral pathway, in which all 150 teleconsultation cases would have been automatically referred for in‐person specialist evaluation and Mpox PCR testing at the tertiary center. Costs were based on standard rates, including specialist consultation (2,500,000 IRR) and Mpox PCR test (7,000,000 IRR). The primary consequences measured were the number of in‐person referrals and PCR tests avoided through remote triage. Data were reported using descriptive statistics (medians, interquartile ranges [IQRs], counts, and percentages).

### 2.6. Ethical Considerations

This study constituted a retrospective evaluation of a public health intervention implemented as part of the mandated outbreak response protocol in Fars Province. As the study analyzed data from a public health surveillance and service delivery activity—not a prospective research intervention with experimental protocols—formal prospective registration with an institutional review board (IRB) was not required under national public health operation guidelines. The intervention itself was authorized and mandated by the Vice Chancellor for Health Affairs at SUMS as part of its statutory duty to respond to public health threats. All patient management followed standard national guidelines and the World Health Organization (WHO) clinical guidelines for suspected Mpox. For this retrospective analysis, all patient data were fully deidentified before review and aggregation. The study protocol for data analysis was reviewed and approved by the relevant administrative authorities at SUMS, confirming that the work adhered to principles of patient confidentiality, data protection, and beneficence as outlined in the Declaration of Helsinki. Informed consent for the teleconsultation was obtained verbally from all patients as part of the standard public health procedure.

## 3. Results

### 3.1. Cohort Characteristics and Flow

During the 28‐day alert period, the provincial telehealth protocol was activated for all patients presenting with fever and vesiculopustular rash. The study cohort consists of 150 patients for whom the local referring physician (Tier 1) could not confidently exclude Mpox, leading to escalation for remote specialist consultation (Tier 2). This cohort did not include patients diagnosed with clear alternative conditions (e.g., typical dermatomal herpes zoster) at the primary care level. The flow of these 150 patients through the triage system is summarized in Figure 1. The median age of the cohort was 32 years (IQR: 18–45), and 92 (61.3%) were male. All patients were Iranian nationals. Three patients (2.0%) reported recent travel to countries with confirmed Mpox cases (i.e., Iraq, Pakistan, and the United Arab Emirates).

### 3.2. Clinical Characteristics of High‐Suspicion Cases

Among the 150 patients managed through the telemedicine protocol, 28 (18.7%) were triaged by the focal points as high suspicion, warranting Mpox PCR testing. Detailed clinical and epidemiological data were available for 22 of these patients (Table 1). The cohort had a median age of 23 years (IQR: 15.75–35.25 and range: 1–77), with 12 (54.5%) being male. The most common occupations were student (27.3%), freelancer (18.2%), and housewife (18.2%). All 22 patients presented with a cutaneous rash, dominantly distributed on the trunk (86.4%) and palms (72.7%). The most common lesion morphology was vesicular (72.7%), with the majority of patients (77.3%) presenting with a monomorphous rash (lesions in the same stage), whereas 22.7% had a polymorphous rash (lesions in different stages). The most frequently reported symptoms were headache (50.0%), fever (45.5%), and myalgia (40.9%). Additionally, lymphadenopathy was present in 22.7% of patients. Six patients (27.3%) required hospitalization. Moreover, regarding epidemiological links, two patients reported close contact with a suspected Mpox case, and three (13.6%) had a history of international travel to countries with confirmed Mpox cases. Notably, patients did not report contact with wild or domesticated animals relevant to Mpox transmission—except one with horse contact.

**TABLE 1:** Clinical and epidemiological characteristics of the 22 high‐suspicion patients who underwent Mpox PCR testing, Fars Province, Iran (2024).

Characteristic	Value (*n* = 22)
Age, years, median [IQR] (range)	23 [15.75–35.25] (1–77)
Sex, %	
Male	12 (54.4)
Female	10 (45.6)
Job, %	
Student	6 (27.3)
Freelancer	4 (18.2)
Housewife	4 (18.2)
Military	3 (13.6)
Health staff	1 (4.5)
Other	4 (18.2)
Reported sign/symptom, %	
Fever	10 (45.5)
Lymphadenopathy	5 (22.7)
Myalgia	9 (40.9)
Back pain	7 (31.8)
Malaise	2 (9.1)
Headache	11 (50.0)
Chills	2 (9.1)
Cough	1 (4.5)
Conjunctivitis	1 (4.5)
Hospitalization required, %	6 (27.3)
Rash distribution, %	
Trunk	19 (86.4)
Palms (hands/feet)	16 (72.7)
Limbs	4 (18.2)
Head and neck	2 (9.1)
Lesion morphology, %	
Vesicular	16 (72.7)
Papular	6 (27.3)
Macular	2 (9.1)
Crust	1 (4.5)
Lesion stage, %	
Monomorphous	17 (77.3)
Polymorphous	5 (22.7)
Epidemiological link, %	
Close contact with a suspected case during the past month	2 (9.1)
International travel history	3 (13.6)

### 3.3. Diagnostic Outcomes and Testing Cascade

Testing was performed selectively based on the teleconsultation outcome. All 28 high‐suspicion patients underwent swab sampling of lesional exudate at the SUMS referral laboratory, with specimens shipped to the Pasteur Institute of Iran for Orthopox PCR testing. All 28 results (100%) were negative for Mpox, including for the three patients with travel history as well as the two patients with suspicious contact history. The final diagnoses for all 150 patients, established via teleconsultation and directed testing, are presented in Table 2. The majority of cases were diagnosed as common viral exanthems; that is, testing for alternative diagnoses showed that 126 patients (84.0%) had confirmatory testing for VZV (PCR/serology) and 12 (8.0%) for HSV, which validated the clinical judgment of the remote specialists. Other infectious dermatoses and noninfectious rashes comprised the remaining eight (5.3%) and four (2.7%) cases, respectively.

**TABLE 2:** Final diagnoses for the entire cohort (*n* = 150).

Final diagnosis	Number of cases (%)
Varicella (chickenpox)	85 (56.7)
Herpes zoster (shingles)	41 (27.3)
Herpes simplex virus infection	12 (8.0)
Other infectious dermatoses	8 (5.3)
Noninfectious rash	4 (2.7)

### 3.4. Efficiency and Time Savings of the Telemedicine System

The asynchronous tele‐triage system directly reduced unnecessary resource utilization. Of the 150 patients, 122 (81.3%) were managed locally following remote specialist advice, avoiding a physical referral to the tertiary center. The median time from case submission by the local physician to receiving structured feedback via the WhatsApp‐based system was 95 min (IQR: 45–180 min). This rapid turnaround enabled timely isolation decisions for high‐suspicion cases and allowed for immediate initiation of appropriate management for others, bypassing delays inherent to travel and scheduling for in‐person consults.

### 3.5. Cost–Consequence Analysis

The cost analysis showed notable savings. Compared to a hypothetical baseline where all 150 patients would have been referred for in‐person specialist consultation and Mpox PCR testing, the implemented pathway resulted in avoidance of 122 unnecessary in‐person specialist consultations, as well as avoidance of 122 unnecessary Mpox PCR tests. The total direct cost of the standard referral pathway was 1,425,000,000 IRR. The cost of the tele‐triage pathway was approximately 337,500,000 IRR (covering 28 in‐person visits and 28 PCR tests). This resulted in net savings of 1,087,500,000 IRR, representing a 76% reduction in direct costs for this cohort. Of note, this calculation does not include additional costs such as transport and hoteling, which can vary.

### 3.6. Representative Case

**Figure 2:**
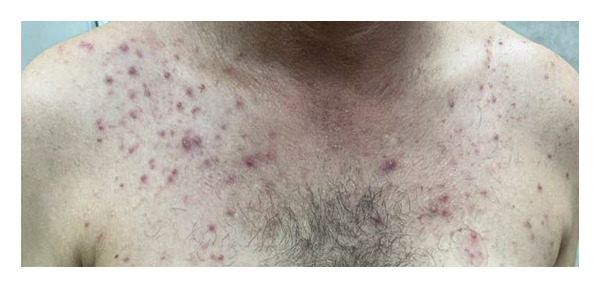
Clinical photograph of a patient evaluated through the 2024 telemedicine triage surveillance protocol. The image shows the truncal rash of a 45‐year‐old man with a history of travel to an Mpox endemic region, who presented with fever and nonpruritic vesiculopustular lesions. This case was managed remotely and was subsequently diagnosed as disseminated herpes zoster following negative Mpox PCR testing, showing the utility of teleconsultation for differential diagnosis.

A representative case was a 45‐year‐old man with a history of recent travel to Karbala, Iraq (i.e., one of the three cases with travel history). He presented with fever and a rash localized to his trunk and arms, without lymphadenopathy. Images and history were sent for teleconsultation (Figure 2). Based on the morphology and distribution, Mpox was considered unlikely, and a diagnosis of disseminated herpes zoster was suggested and later confirmed, which prevented an unnecessary referral and PCR test. Currently, there are no fever and itchiness.

## 4. Discussion

Our study found that a mandated tiered hub‐and‐spoke telemedicine model was able to effectively triage 150 suspected Mpox cases during a 28‐day provincial alert in a setting with no confirmed local transmission. The system successfully ruled out Mpox in 122 patients (81.3%) via remote consultation, which enabled local management and averted unnecessary referrals to a tertiary center. Additionally, all 28 high‐suspicion cases tested for Orthopoxvirus PCR were negative, with final diagnoses involving common viral exanthems (i.e., varicella zoster virus and herpes simplex virus infections). Furthermore, this operational outcome coupled with a median specialist response time of 95 min and a 76% reduction in direct costs collectively underpinned the model’s utility as a high‐efficiency filter for outbreak surveillance.

The success of this model depended on its operational design. Decentralizing initial assessment to local spoke physicians leveraged frontline access, while centralizing expert review at the hub optimized the use of scarce infectious disease expertise in the region. The high negative predictive value of remote triage is notable; that is, specialists confidently excluded Mpox for the vast majority based on history and imagery, a judgment that was validated by molecular or serological testing for alternative pathogens (VZV/HSV). Such diagnostic accuracy is favorable in a high‐alert, low‐prevalence scenario, where the primary challenge is distinguishing Mpox from common mimickers [28, 29]. The rapid median response time facilitated timely isolation decisions for the few high‐suspicion cases, allowed for immediate initiation of appropriate care for others, and bypassed delays inherent to travel and in‐person consultation.

From a health system perspective, our model functioned as an infection prevention and control (IPC) strategy by minimizing unnecessary physical contact and potential exposure events in healthcare settings [22]. Moreover, the model’s efficiency was translated into substantial economic benefit, using a simple economic model. The avoided costs of 122 specialist consultations and 122 unnecessary Mpox PCR tests represent a conservative estimate of savings, as patient transportation, lodging, and lost productivity were not included. This 76% cost reduction might highlight the model’s role not merely in saving money but in enabling more rational resource allocation. Accordingly, it has also been reported that telemedicine can reduce the financial burden on health systems during infectious disease outbreaks by minimizing in‐person referrals and associated costs [15]. Moreover, by preventing a surge of low‐yield referrals, the system protected the tertiary hospital and reference laboratory capacity for genuine emergencies. Therefore, one may suggest the approach as a scalable blueprint for managing initial alerts for other emerging infectious diseases with characteristic dermatological presentations (e.g., novel viral exanthems)—or specific presentations in general—in similar resource‐limited settings.

Our findings contribute to the growing literature on telemedicine for Mpox, but with a distinct focus on system‐wide frontline triage. Previous reports have shown its value in follow‐up care for confirmed cases, monitoring contacts, or delivering therapeutics. For example, Siciliano et al. [30] showed its utility in clinical follow‐up and treatment during the Mpox outbreak in Ibiza and Formentera, ensuring patient safety and adherence to isolation protocols. Additionally, Seah et al. [31] found its role in monitoring close contacts and assessing postexposure prophylaxis, although there were challenges in evaluating sensitive anatomical areas. Moreover, Chan et al. [21] reported the scalability of telehealth models in rapidly expanding treatment capacity during the 2022 Mpox outbreak in New York City, particularly in adhering to CDC protocols for tecovirimat administration. Our study showed its role in the initial gatekeeping phase of an outbreak response. Among other successful applications reported during the Mpox outbreak, Warrell et al. [32] implemented a virtual ward in the United Kingdom to manage confirmed Mpox cases, where they safely enabled outpatient care for 86% of patients and averted hospital admissions. It should be noted that although our model focused on filtering referrals to preserve specialist and laboratory capacity, the virtual ward model focused on preserving inpatient bed capacity. Both applications address core health system constraints during outbreaks. Furthermore, the 81.3% referral avoidance rate was in line with established evidence from SAF teledermatology [19, 31, 33–35], allowing clinicians to manage more patients simultaneously. A comprehensive review of SAF practices reported that 23%–84% of consultations can be managed remotely, depending on the setting and whether images are taken by clinicians or patients [35]. Liddy et al. [35] found that only 18% of cases required in‐person consultations after asynchronous triage, compared to 50% under previous systems. Similarly, other studies have shown that 68% of in‐person dermatology appointments were unnecessary when asynchronous teledermatology was employed [19, 36]. Moreover, studies indicated that asynchronous dermatology consultations take as little as 4 min, compared to traditional in‐person visits, which optimizes system throughput and reduces unnecessary referrals. Such accordance with dermatologic telemedicine between our study and the just‐mentioned studies is intuitive, given the centrality of cutaneous findings. In the context of Mpox, where cutaneous findings are critical for diagnosis, asynchronous consultations were able to minimize referrals, reduce transmission risks, and decrease healthcare expenditures. Our experience confirmed that these principles are transferable, adaptable, and effective in the acute public health context of infectious disease surveillance [22].

Our study has several limitations. First, the cohort represented only cases that primary care could not rule out, creating a selection bias. We could not calculate the sensitivity or specificity of the overall surveillance system, as we lack data on all patients presenting with fever and rash during the alert period. Second, the model’s effectiveness depended on a pre‐existing, engaged network of physicians and specialist buy‐in; that is, its replicability in settings without such foundational coordination may vary. Third, although WhatsApp provided accessibility and speed, its use raises questions about compliance with formal data security and privacy standards (e.g., HIPAA and GDPR). The implementation of telemedicine systems should consider a secure infrastructure, which can be a challenge. Fourth, our cost–consequence analysis adopted a health system payer perspective; a societal perspective including patient costs might show even greater savings. Finally, the model’s performance was evaluated during a short, specific alert for Mpox, which has a relatively low case fatality rate [37]; hence, its sustainability and effectiveness during outbreaks of pathogens with higher severity or longer duration warrant further evaluation. Worth noting, key strengths of the study included the real‐world and protocol‐mandated implementation, the structured hub‐and‐spoke design, and the cost analysis from a health system perspective.

## 5. Conclusion

The implementation of a tiered telemedicine model during a high‐alert Mpox period in Southern Iran led to the efficient triage of 150 suspected cases, accurately identified common viral exanthems, and prevented over 80% of unnecessary specialist referrals, totally resulting in significant resource savings. This approach represented coordinated and asynchronous telemedicine as a scalable tool for frontline outbreak surveillance in high‐alert, low‐prevalence settings. Future health systems may consider adopting similar hub‐and‐spoke models for alert management, with attention to secure communication and network training.

## Author Contributions

O.E. and N.E. contributed substantially to the design of the study; O.E., N.E., M.N., and M.S. acquired the data; A.H. curated the data; M.S. and A.H. had roles in data interpretation; A.H. prepared the visualizations; O.E., A.H., and N.E. wrote the initial draft; and all the authors reviewed the manuscript critically.

## Funding

The authors received no specific funding for this work.

## Conflicts of Interest

The authors declare no conflicts of interest.

## Data Availability

The data that support the findings of this study are available on request from the corresponding author. The data are not publicly available due to privacy or ethical restrictions.
